# Defining the learning curve for thulium Laser *en bloc* resection of bladder tumors: a single-surgeon retrospective cohort study

**DOI:** 10.3389/fsurg.2026.1769917

**Published:** 2026-02-24

**Authors:** Xiaodong Qing, Xiangzheng Wu, Wenbo Gao

**Affiliations:** 1 Department of Surgery, Ningbo Urology and Nephrology Hospital, Ningbo, Zhejiang, China; 2 Department of Urology, Ningbo Urology and Nephrology Hospital, Ningbo, Zhejiang, China

**Keywords:** bladder cancer, *en bloc* resection, learning curve, retrospective, thulium laser

## Abstract

**Objective:**

To quantitatively analyze the learning curve for thulium laser *en bloc* resection of bladder tumor (TL-ERBT) performed by a surgeon experienced in conventional transurethral resection of bladder tumor (TURBT).

**Methods:**

In this single-surgeon, retrospective cohort study, the initial 86 consecutive TL-ERBT cases were reviewed. Operation time was used as the primary outcome. Learning curve analysis was performed using moving average and cumulative sum (CUSUM) methods.

**Results:**

Among 79 successfully completed TL-ERBTs, the mean operation time was 31.6 ± 10.3 min. CUSUM analysis identified a turning point at case 32, separating the Learning phase (cases 1–32) from the Proficiency phase (cases 33–86). Operation time significantly decreased from 37.8 ± 9.2 min in the Learning phase to 27.8 ± 8.1 min in the Proficiency phase (*P* < 0.001). The conversion rate to conventional TURBT declined from 12.5% to 2.1% (*P* = 0.038). Detrusor muscle presence in specimens (87.3% overall) and major complication rates were comparable between phases.

**Conclusion:**

For a surgeon experienced in conventional TURBT, preliminary evidence from this study suggests that proficiency in TL-ERBT, defined primarily by operative efficiency, may be achievable after approximately 32 procedures, with significant improvements in operative efficiency and technical success; while patient safety was not compromised. These findings provide a practical quantitative benchmark for surgical training and clinical implementation.

## 1 Introduction

Bladder cancer (BC) is the sixth most common cancer in men and seventeenth most common cancer in women worldwide (1). It is also the second most common genitourinary tract malignancy worldwide, with non-muscle-invasive bladder cancer (NMIBC) accounting for about 70%–80% of new cases. Nowadays, transurethral resection of bladder tumor (TURBT) remains the standard treatment of NMIBC. However, conventional TURBT, which employs monopolar or bipolar piecemeal resection, has inherent limitations. These include risk for obturator nerve stimulation, thermal damage to surrounding tissues, and most critically, potential tumor residue due to fragmented tissue, which compromise accurate pathological staging and increase recurrence rates (2).

In recent years, *en bloc* resection of bladder tumor (ERBT) has emerged as a promising alternative (3). This technique aims to remove the tumor together with the underlying detrusor muscle as an intact specimen. It provides potential benefits for complete histopathological evaluation, such as clear margin assessment and precise staging, as well as reducing recurrence (4). Various energy platforms, including holmium and thulium lasers, have been adapted for ERBT. The thulium laser operates in continuous wave mode at a wavelength near the water absorption peak (1.75–2.22 μm, with an average of 1.940 μm). It provides excellent hemostatic and precise cutting characteristics, making it theoretically eligible for the meticulous anatomical dissection required in ERBT (5).

While the feasibility and short-term efficacy of thulium laser ERBT (TL-ERBT) have been documented in the literature, its widespread clinical adoption remains limited (6). An important reason is the procedural complexity involved in achieving ideal resection quality, which demands precise identification of surgical anatomy and tissue planes. Moreover, the learning curve associated with this technique remains poorly defined.

In surgical fields, the learning curve is generally understood as the initial phase during which a procedure is less efficient, more technically challenging, and associated with higher complication rates due to the surgeon's lack of experience. As noted by Alexander K. et al., it refers to the time required for a surgeon to perform the procedure within a reasonable operative duration while achieving acceptable complication rates, along with satisfactory oncological and functional outcomes (7). In practice, the implementation and outcomes of a surgical technique are influenced by several critical factors, including surgeon-related variables such as attitude, confidence, and prior operative experience, as well as team-related variables. This underscores the importance of understanding the learning curve, as it directly impacts procedural efficiency and patient safety (8).

To date, only a few studies have analyzed the learning curve for conventional TURBT (9), and data on TL-ERBT are even more scarce. This lack of related data makes it difficult to set realistic standards for proficiency and design effective training protocols for this novel laser technique. Consequently, it is crucial to quantify the learning curve of TL-ERBT for its safe, rational implementation and effective training in clinical practice.

To address this knowledge gap, we conducted this retrospective study analyzing the initial experience at our institution. The operating surgeon in this study (Dr. W.G.) is an expert experienced in conventional TURBT, having performed over 300 procedures, but was novice to TL-ERBT. After comprehensive self-preparation through literature and operational video observation, he independently underwent TL-ERBT. The objective of this cohort study was to evaluate the surgical learning curve of TL-ERBT in the initial 86 cases. The primary surrogate endpoint for learning curve assessment was operation time. Secondary endpoints included the rate of conversion to conventional TURBT and the presence of detrusor muscle in the resection specimen. This study aimed to provide empirical data to guide training and implementation protocols.

## 2 Materials and methods

### 2.1 Study design and patient selection

A retrospective single-center cohort study was conducted, analyzing the initial 86 consecutive cases of TL-ERBT performed by a single senior surgeon (Dr. W.G.) from January 2022 to September 2025. In this study, all patients had pathologically confirmed non-muscle-invasive bladder cancer (NMIBC) and underwent ERBT as primary treatment. Seven cases that required conversion to conventional TURBT due to technical difficulties or safety concerns were excluded from the learning curve analysis for operation time. Exclusion criteria also included muscle-invasive bladder cancer (MIBC), salvage procedures, or with incomplete clinical data. Additionally, patients with more than 4 bladder tumors were excluded. This threshold was set based on current literature indicating that tumor multiplicity remains a significant technical constraint for ERBT, with many experts suggesting that excessive tumor numbers (>4) may compromise the feasibility and oncological completeness of *en bloc* resection (3, 10). By limiting the cohort to patients with ≤4 tumors, our study aimed to evaluate the learning curve under conditions that reflect the current consensus on feasible ERBT indications. Of the initial 86 intended cases, the main reasons for exclusion from the final analysis of successfully completed TL-ERBT (*n* = 79) were: conversion to conventional TURBT (*n* = 7). Among the converted cases, reasons included persistent bleeding obscuring visualization (*n* = 4) and tumor location (anterior wall/dome) posing excessive technical challenge early in the series (*n* = 3). All patients gave written informed consent and the study was approved by the Institutional Ethics Committee of our hospital in compliance with the Declaration of Helsinki.

### 2.2 Data collection and statistical analysis

Patient demographic and clinical characteristics included age, gender, tumor number (categorized as single vs. multiple), maximum tumor diameter and tumor location (categorized as lateral wall, posterior wall, trigone, dome, or anterior wall). Pathological evaluation of the resection specimen included assessment for the presence of detrusor muscle- a key indicator of resection quality and staging. Operation time was defined as the duration from resectoscope insertion to complete tumor resection and hemostasis. Conversion rate to conventional TURBT was also recorded.

Statistical analyses were performed with the statistical software program SPSS (SPSS Inc., Chicago, IL, version 20.0) for Windows. Continuous variables were expressed as mean ± standard deviation, and compared using independent *t*-tests for normally distributed data or Mann–Whitney U tests for non-normally distributed data after assessing normality with Shapiro–Wilk tests. Categorical variables were expressed as counts (percentages), and compared using chi-square tests or Fisher's exact tests when expected cell counts were less than 5. Statistical significance was set at *P* < 0.05.

### 2.3 Learning curve analysis

The operation times of all patients who successfully completed ERBT were collected chronologically, from the earliest to the latest date of operation. The primary outcome measure for learning curve analysis was operation time. Two complementary statistical methods were employed:

#### 2.3.1 Moving average method

A 10-case moving average was calculated to smooth the operative time sequence data and visualize overall trends.

#### 2.3.2 Cumulative sum (CUSUM) analysis

**Figure 1:**
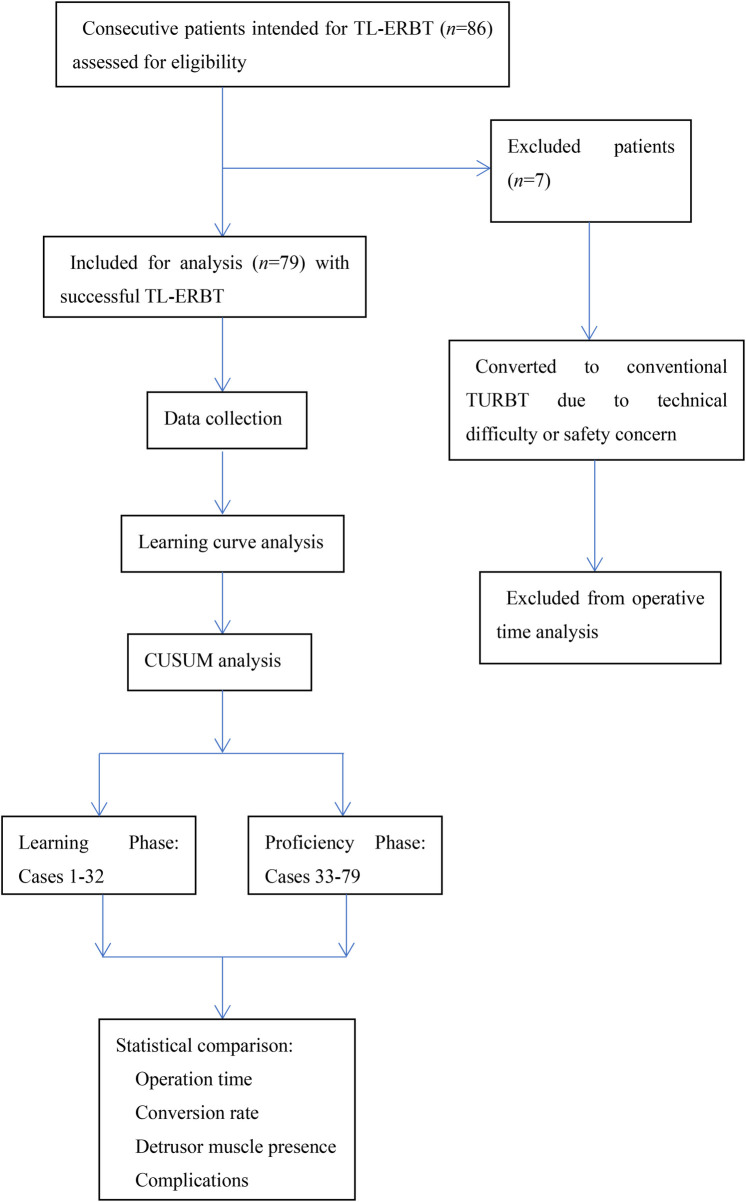
Study flow chart.

CUSUM values were calculated using the formula: CUSUMi = (Xi - *μ*) + CUSUMi-1, where Xi represents the operation time for case i, and μ is the mean operation time for all 79 successfully completed ERBT cases. The peak of the CUSUM curve was identified as the turning point, dividing cases into Learning phase and Proficiency phase. The study flow chart is seen in Figure 1.

## 3 Surgical technique

During the operation, the patient was in the lithotomy position under general or continuous epidural anesthesia, using a 26 F continuous-flow resectoscope with normal saline irrigation. A thulium laser system (Vela XL, Boston Scientific Corporation) was employed, with the laser power typically set at 25–30 W, which was adjusted according to the volume of the tumor and the site of the surgical resection (11).

The *en bloc* resection technique was performed as follows: After complete cystoscopic inspection to assess tumor number, size and location, the resection commenced with a circumferential mucosal incision using the laser fiber. This incision was made approximately 0.5–1.0 cm from the visible tumor border as a safety margin. The resection then proceeded deeper into the muscular layers. Using a combination of precise laser cutting and blunt dissection with the resectoscope tip, the plane between the tumor base (including a portion of the underlying detrusor muscle) and the deeper bladder wall was developed. This “traction-and-countertraction” technique helped expose the dissection plane and minimize risk of perforation. Hemostasis for small vessels was achieved simultaneously by use of the coagulation property of the laser.

For tumors in difficult locations, such as the anterior wall or dome, technical adjustments were made. The bladder was partially filled with irrigation fluid, and an assistant applied suprapubic pressure to improve exposure. Resection in these areas often proceeded from the lateral aspects towards the center, with careful lifting of the tumor base using the sheath, so as to maintain visualization and avoid inadvertent deep injury. For tumors adjacent to the ureteral orifice, a ureteral stent (e.g., double-J stent) was placed prophylactically to protect the orifice and facilitate identification.

**Figure 2:**
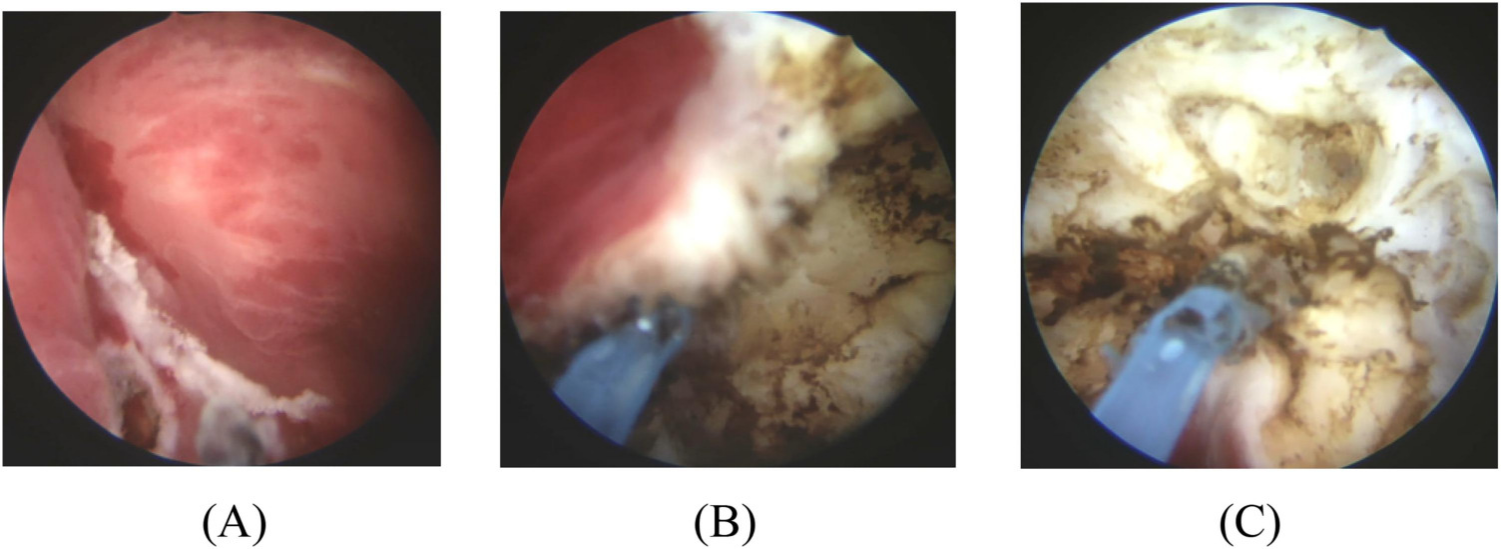
Surgical steps of TL-ERBT. **(A)** Circumferential incision around the tumor with a safety margin of 5–10 mm. **(B)** Dissection proceeding into the deep muscular layer under direct vision. **(C)** *En bloc* specimen of almost complete resection.

The final crucial step was to extract the intact specimen. For specimens estimated to be smaller than 3 cm in greatest dimension, extraction was usually accomplished by flushing the specimen through the resectoscope sheath or an Ellik evacuator. For specimens between 3 and 4 cm, we generally retained the outer sheath of the resectoscope and flushed the specimen out as per the siphon effect (12). The surgical procedures are illustrated in Figure 2.

Upon completion, the tumor bed and edges were carefully inspected and any remaining bleeding points were coagulated. A 20F Foley catheter was then inserted for postoperative drainage and irrigation.

## 4 Results

### 4.1 Learning curve analysis

The mean operation time for the entire cohort of 79 patients who successfully completed TL-ERBT was 31.6 ± 10.3 min. The learning curve was assessed using two complementary statistical methods: the moving average analysis and CUSUM analysis.

### 4.2 Moving average analysis

**Figure 3:**
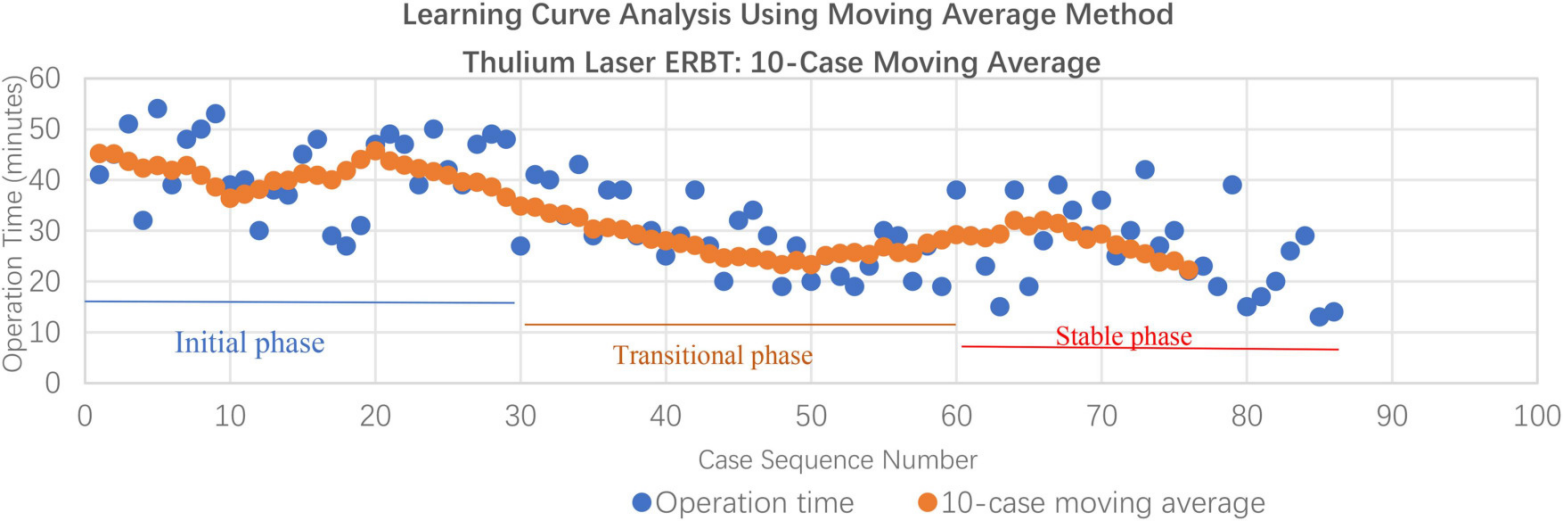
10-case moving average of operation time for TL-ERBT procedures, illustrating the learning curve trend across sequential cases.

The 10-case moving average of operation time was plotted sequentially to illustrate the trend with case-by-case variability, as seen in Figure 3. The curve demonstrated three distinct phases: an initial high-variability phase (first 30 cases) with average times of 35–45 min, a transitional phase (cases 30–60) with gradual reduction to 30–35 min, and a stable phase (after case 60) with consistent times of 25–30 min.

### 4.3 CUSUM analysis

**Figure 4:**
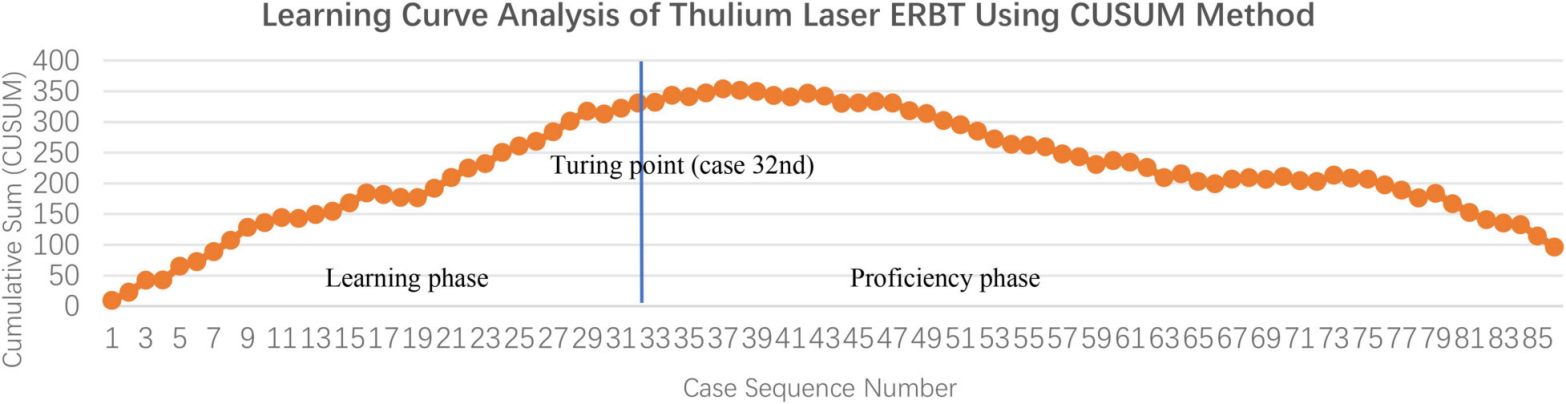
Cumulative sum (CUSUM) analysis of operation time for TL-ERBT, identifying the turning point at case 32 as the transition from learning to proficiency phase.

CUSUM analysis of operation time revealed a distinct turning point at case 32nd, establishing this as the threshold between the initial learning phase and subsequent proficiency phase (Figure 4). This division was further supported by the moving average analysis, which showed stabilization of operation times after approximately 30 cases.

Consequently, cases 1 to 32 were classified as the Learning phase and cases 33 to 86 as the Proficiency phase for all subsequent comparative analyses.

Herein, Table 1 summarizes and compares the operation time characteristics resulting from both the moving average analysis and the CUSUM-determined phase division.

**Table 1:** Operation time characteristics across different phases of the TL-ERBT learning curve.

Phase definition	Case range	Mean operation time (min)	Time range (min)	Comments
Moving average analysis				
Initial phase	1–30	∼40*	35–45	Marked by inconsistent performance.
Transitional phase	31–60	∼32.5*	30–35	Gradual improvement and stabilization.
Stable phase	61–86	∼27.5*	25–30	Consistent and efficient performance.
CUSUM analysis				
Learning phase	1–32	37.8 ± 9.2	–	Turning point identified at Case 32.
Proficiency phase	33–86	27.8 ± 8.1	–	Significant reduction (*P* < 0.001).

* Values denoted with “∼” were approximate means deriving from the moving average trend description in the text.

### 4.4 Conversion to conventional TURBT

A total of 7 cases (8.1%) required conversion to conventional TURBT during the operation. The most common reasons for conversion were intraoperative bleeding obscuring visualization (*n* = 4) and tumors located in technically challenging positions (anterior wall: *n* = 2; dome: *n* = 1). The distribution of conversions across learning curve showed a clear declining trend: 4 conversions occurred in the learning phase (4/32, 12.5%), 2 in the transitional period (cases 33–38), and 1 in the subsequent stable period (cases 39–86). Overall, the conversion rate decreased significantly from 12.5% in the Learning phase to 5.6% in the Proficiency phase (cases 33–86, 3/54; *P* = 0.038). This reflects improved technical facility as experience accumulated (Table 2).

**Table 2:** Patient characteristics and operative outcomes in learning phase and proficiency phase.

Variable	Learning phase (cases 1–32)	Proficiency phase (cases 33–86)	*P*-value
Age (years), mean ± SD	67.8 ± 8.3	68.9 ± 9.1	0.564
Gender			0.917
Male	32 (84.2%)	40 (83.3%)	
Female	6 (15.8%)	8 (16.7%)	
Maximum tumor diameter (cm), mean ± SD	1.75 ± 0.75	1.74 ± 0.76	0.95
Tumor location, *n* (%)			0.621
Lateral wall	18 (47.4%)	25 (52.1%)	
Posterior wall	9 (23.7%)	8 (16.7%)	
Trigone	5 (13.2%)	8 (16.7%)	
Dome	3 (7.9%)	4 (8.3%)	
Anterior wall	3 (7.9%)	3 (6.3%)	
Operation time (minutes), Mean ± SD	37.8 ± 9.2	27.8 ± 8.1	<0.001
Tumor number			0.856
Single	33 (86.8%)	41 (85.4%)	
Multiple (2–4)	5 (13.2%)	7 (14.6%)	
Conversion to TURBT, *n* (%)^a^	4 (12.5%)	3 (5.6%)	0.038
Detrusor muscle presence, *n* (%)^b^	29 (85.3%)^c^	40 (88.9%)^c^	0.745
Major complications, *n* (%)^b^	2 (5.3%)^c^	1 (2.1%)^c^	0.723

^a^ The “Proficiency Phase” in this table was Cases 33–86, which included the transitional period (Cases 33–38) and the subsequent stable proficiency period. The conversion rates were therefore calculated as 4/32 (12.5%) for the Learning Phase and 3/54 (5.6%) for the combined Proficiency Phase.

^b^ Major complications included: postoperative bleeding requiring surgical re-intervention, bladder perforation requiring prolonged catheterization or repair, or sepsis.

^c^ Denominators for these outcomes referred to the number of cases successfully completed with TL-ERBT in each phase (i.e., excluding cases converted to conventional TURBT).

### 4.5 Patient characteristics and operative outcomes

A comparison of patient demographics, tumor characteristics, and operative outcomes between the two phases is summarized in Table 2.

No significant differences were observed between the Learning phase and Proficiency phase with respect to patient age (67.8 ± 8.3 years vs. 68.9 ± 9.1 years, respectively; *P* = 0.564) or gender distribution (84.2% male *vs.* 83.3% male; *P* = 0.917). Tumor number (single *vs.* multiple) was also comparable between the two groups (86.8% single *vs.* 85.4% single; *P* = 0.856). Similarly, no statistically significant difference was observed in maximum tumor diameter, with tumors resected during the Learning phase being 1.75 ± 0.75 cm on average and 1.74 ± 0.76 cm in the Proficiency phase (*P* = 0.95). Tumor location distribution is detailed in Table 2. In the surgeon's experience, tumors on the anterior wall and dome were associated with greater technical difficulty, occasionally leading to longer operative times or conversion, especially during the early learning phase.

Most notably, a significant reduction in operation time was observed after the learning curve transition. The mean operation time decreased from 37.8 ± 9.2 min in the Learning phase to 27.8 ± 8.1 min in the Proficiency phase (*P* < 0.001), representing a mean reduction of 10.0 min (26.5%).

### 4.6 Clinical outcomes

Of the 86 intended TL-ERBT procedures, 79 (91.9%) were completed successfully without conversions to conventional TURBT. Neither conversion to open surgery nor perioperative mortality occurred in all patients.

**Figure 5:**
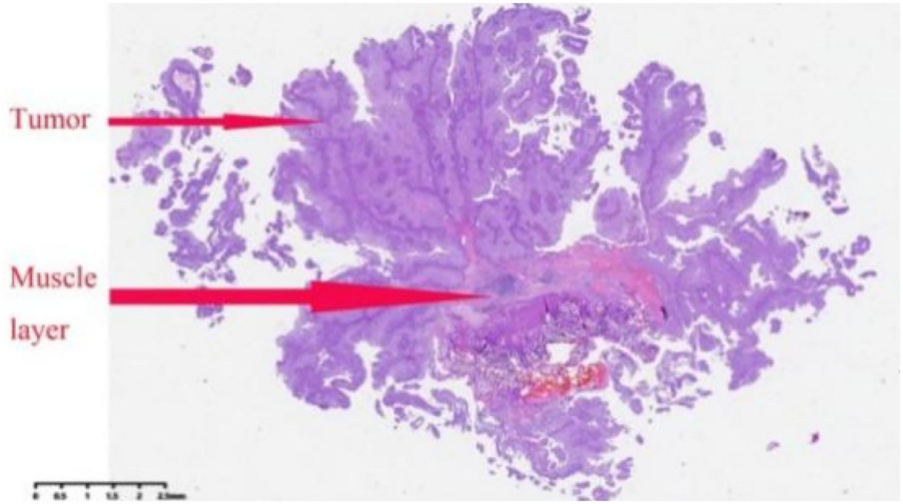
A histopathological image of TL-ERBT specimen (H&E staining, 2 × 10).

Detrusor muscle was identified in the pathological specimen in 87.3% of overall cases (69/79). The presence rate was comparable between the Learning phase and the Proficiency phase: 85.3% (29/34) vs. 88.9% (40/45), respectively (*P* = 0.745). This suggests that even during the initial learning period, the surgeon was able to achieve adequate resection depth for pathological staging in the majority of cases. (A histopathological image of TL-ERBT specimen is shown in Figure 5).

As shown in Table 2, the overall safety of the procedure was favorable. The incidence of major complications (defined as postoperative bleeding requiring surgical re-intervention, bladder perforation requiring prolonged catheterization or repair, or sepsis) was low; and there was no statistically significant difference between the Learning phase (2/38, 5.3%) and the Proficiency phase (1/48, 2.1%) (*P* = 0.723).

## 5 Discussion

This study objectively evaluates the learning curve for TL-ERBT performed by a surgeon experienced in conventional TURBT through self-directed learning. Our main finding is that a clear learning curve exists, with proficiency- defined by a significant and sustained reduction in operation time- achieved after approximately 32 procedures. It is important to clarify that operation time serves as a surrogate for technical efficiency in this learning curve analysis. While it is an objective and widely used metric, it does not directly measure oncological adequacy or long-term outcomes, which should be evaluated separately.

The surgeon's prior experience with over 300 conventional TURBT procedures, in combination with preparatory literature review and video observation, likely provided a critical foundation. This background may have allowed for a faster understanding of bladder anatomy required for ERBT, and main goals of tumor resection. Therefore, our results showed that the resection method could shift more efficiently towards the novel techniques of instrument handling (laser control) and precise resection (*en bloc* resection).

The Learning phase (first 32 cases) was characterized by longer and more variable operation times (mean 37.8 min). CUSUM analysis identified case 32nd as the definitive turning point. After this point, operation times stabilized at a significantly lower level- mean 27.8 min- in the Proficiency phase, with a 26.5% reduction. This trend was visually supported by the moving average analysis. The number of cases needed to overcome the learning curve in our study (32 cases) is consistent with other reports for laser-based *en bloc* techniques. For example, in a recent prospective study by Yao et al. (13) who used an *ex vivo* porcine model, the learning curve for ERBT was quantified. Their CUSUM result indicated that the range varied between 20 and 40 procedures, which underscored a reliable pattern for acquiring proficiency. Therefore, this technique appears particularly attainable when the surgeons, as in our study and Yao's (13), possess relevant operative skills and undertake dedicated preparatory theoretical study.

Furthermore, our results demonstrated that the learning process was associated with improved operative skill, as reflected by a significant decline in the conversion rate to conventional TURBT (from 12.5% in the Learning phase to 5.6% in the Proficiency phase). The most common reasons for conversion were bleeding impairing visualization and challenging tumor locations (anterior wall/dome). This reduction confirmed the surgeon's increasing mastery of laser properties, precision in anatomical dissection, and ability to manage tumors in different locations. The conversion rate also serves as a recognized indicator of intraoperative technical difficulty or safety concerns (14). Importantly, this improvement occurred despite comparable patient demographics and tumor characteristics between the two phases, suggesting that progress stemmed primarily from enhanced surgical skill rather than selection bias toward simpler cases.

Given the scarcity of clinical studies directly evaluating the learning curve for TL-ERBT, insights can be drawn from analogous laser enucleation procedures in urology, such as holmium laser enucleation of the prostate (HoLEP). In a comparative study by Kim et al. (15), the authors reported that beginner urologists could achieve proficiency in resection speed for HoLEP after approximately 16 cases, indicating that the perceived steep learning curve of laser enucleation may be mitigated through structured experience. Additionally, a survey from high-volume training centers suggested that performing more than 25 cases enabled surgeons to confidently master key technical steps in HoLEP (16). Thus, consistent with findings from laser prostate surgery, the accumulation of surgical experience appears essential to mastering such laser-based techniques.

Notably, the quality of resection, as reflected by the presence of detrusor muscle in the specimen, remained consistently high throughout the learning process (87.3%), with no statistically significant difference between the Learning and Proficiency phases. This aligns with findings from previous studies on laser ERBT, which reported high detrusor identification rates (often >85%) (17); and highlights one of the technique's principal advantages: the ability to provide well-oriented, intact specimens for accurate pathological evaluation (12).

Safety is an important consideration in the adoption of any novel surgical technique. In our series, patient safety was not compromised throughout the learning period. The occurrence of major complications remained low, with 2 and 1 cases in the Learning and Proficiency phases, respectively. This finding is consistent with the safety of laser-based *en bloc* resection reported in the literature. For instance, a prospective study on thulium laser ERBT (TmL-ERBT) reported no instance of obturator nerve reflex or bladder perforation among 23 patients, with only 1 case (4.3%) of clot retention (18). Similarly, a systematic review and meta-analysis comparing thulium laser resection with conventional TURBT showed significantly fewer intraoperative complications, such as obturator nerve reflex (RR 0.04, *p* < 0.001) and bladder perforation (RR 0.11, *p* < 0.001), in the laser group (19). Moreover, recent clinical studies have also affirmed the safety and efficacy profile of thulium laser in urological procedures, supporting its application in ERBT (20, 21). These outcomes demonstrated that TL-ERBT carries an inherent safety advantage, largely attributable to the precise cutting properties of thulium laser energy and the absence of electrical current (11). Our experience further corroborates that, through appropriate patient selection and meticulous technique, TL-ERBT can be adopted safely even during a surgeon's early learning curve. Importantly, there were no conversions to open surgery or perioperative deaths in our cohort. This reinforces the reliable safety of the procedure. Thus, while the learning curve influences operative efficiency and technical success rates, it does not appear to adversely impact fundamental patient safety. And we believe that it is a crucial reassurance for urologists transitioning to this advanced endoscopic approach.

This study has several limitations. First, its retrospective, single-center design and data from a single surgeon limit the generalizability of the findings. The learning curve may vary for surgeons with different levels of skill or in different institutional settings. Additionally, the surgeon's extensive prior experience likely shortened the learning curve, and these results may not apply to novices. Second, while operation time is a robust and objective metric for learning curves, other important outcomes, such as long-term oncological results, were not analyzed and should be the focus of future research. Third, excluding patients with more than 4 bladder tumors may have simplified the initial experience, potentially influencing the learning curve. Fourth, regarding the assessment of data distribution for selecting parametric or non-parametric tests, we relied primarily on the Shapiro–Wilk test. Supplementary visual assessments (e.g., Q-Q plots) could provide additional insight, and their absence is a methodological limitation of our statistical analysis.

## 6 Conclusions

This single-surgeon retrospective analysis provides preliminary evidence of a learning curve for TL-ERBT. For a surgeon with substantial prior experience in conventional TURBT, proficiency, primarily defined by operative efficiency, may be achievable after approximately 32 procedures. Operation time decreased significantly by a mean of 10.0 min (26.5%) after the learning phase, while technical feasibility improved, as reflected by a marked reduction in conversion rates. Safety was maintained throughout, with no significant difference in major complications between phases. However, these findings, derived from a retrospective, single-surgeon series, should be interpreted as preliminary benchmarks rather than definitive standards.

These findings offer a practical benchmark for surgical training: urologists experienced in conventional TURBT can anticipate a learning period of about 30–35 cases, during which operative efficiency and technical success steadily improve. Initiating practice with solitary, smaller tumors in favorable locations may help reduce early conversion rates and smooth the learning transition.

## Data Availability

The raw data supporting the conclusions of this article will be made available by the authors, without undue reservation.
